# The feasibility and efficacy of the group-based therapy for smoking cessation in Klang Valley, Malaysia

**DOI:** 10.18332/tid/175617

**Published:** 2024-03-12

**Authors:** Pakri Mohamed Rashidi Mohamed, Farizah Mohd Hairi, Christopher Bullen, Amer Siddiq Amer Nordin

**Affiliations:** 1 Department of Psychological Medicine, Faculty of Medicine, University of Malaya, Kuala Lumpur, Malaysia; 2 Department of Family Medicine, Faculty of Medicine, The National University of Malaysia, Kuala Lumpur, Malaysia; 3 Department of Social and Preventive Medicine, Faculty of Medicine, University of Malaya, Kuala Lumpur, Malaysia; 4 School of Population Health, Faculty of Medical and Health Sciences, University of Auckland, Auckland, New Zealand

**Keywords:** cigarette, smoking, cessation, group-based therapy

## Abstract

**INTRODUCTION:**

Tobacco epidemic is a global public health concern, killing more than 8 million people annually. Individual therapy is the standard of care of behavioral intervention for smoking cessation in Malaysia and group-based therapy for smoking cessation is an alternative to behavioral intervention commonly used in the western population effectively. The study explored the feasibility and efficacy of group-based therapy for smoking cessation for smokers who want to quit smoking at a quit smoking clinic and community centers in an urban setting in Malaysia.

**METHODS:**

A total of 40 participants who were active smokers and fulfilled the criteria were recruited for the study at the quit smoking clinic. Participants were given behavioral support based on the GBT-M module and individually for 7 weeks with both groups receiving behavioral intervention plus pharmacotherapy.

**RESULTS:**

The median age of participants was 48 years for individual therapy and 45 years for group therapy. Group-based therapy was comparable to individual therapy in smoking abstinence outcome at 4 weeks post quit date (35% vs 30%).

**CONCLUSIONS:**

Group-based therapy was equally effective compared to individual therapy, similar to the western population. Using a group format should allow more people to be treated by a therapist, and therefore could be more cost-effective. Group-based therapy is an option to be included as part of the smoking cessation program in Malaysia.

## INTRODUCTION

In 2020, 22.3% of the global population used tobacco, 36.7% of all men and 7.8% of the women. Tobacco kills more than 8 million people each year with more than 7 million of those deaths being the result of direct tobacco use while around 1.2 million are the result of non-smokers being exposed to secondhand smoke. It was estimated that over 80% of the world’s 1.3 billion tobacco users live in low- and middle-income countries. Globally, tobacco control efforts in both prevention and treatment have advanced in the past 20 years^1^ with the introduction of policies such as generational endgame and MPOWER by Framework Convention for Tobacco Control World Health Organization (WHO). The behavior of tobacco uptake and consumption is encompassed within various social, environmental, cultural and economic factors and impact people in all age groups. Factors, including sex, age, education level, and income are important predictors of all forms of tobacco use. In addition to policies, smoking cessation interventions which highlight cost-effective and safer interventions such as minimal interventions (providing self-help materials) up to intensive intervention by providing medication to assist smokers to quit smoking, were routinely offered^2^. In the end, the attempt of quitting cigarette smoking depends on the individuals who smoke. Despite all these efforts, the majority of smokers fail their quit attempt largely because of their dependence on nicotine and non-nicotine sensory and behavioral cues that reinforce their smoking behaviour^3^.

A variety of behavior therapies, ranging in complexity from simple advice offered by a physician, or other healthcare provider, to much more extensive therapies, are efficacious for cigarette smoking cessation. The success rate for abstinence from cigarette smoking increases when behavioral therapy is combined with pharmacotherapy. Psychotherapy is a type of therapy that is used to treat issues related to emotions and mental health conditions^4^. Psychotherapy is the informed and intentional application of clinical methods and interpersonal stances derived from psychological principles to assist people to modify their emotions, behavior, and other personal characteristics in the direction of the desired changes^5^. Individual therapy is defined as a therapeutic session involving one patient and at least one therapist. A group-based therapy is known as having more than one patient in one session with at least one therapist, and the size of the group therapy depends on the type of therapy the patients are having^6^. A behavioral intervention involves discussion, encouragement, advice, and other modalities to help behavioral change^7^.

Intensive group therapy is a form of psychological treatment where a small group of patients meets regularly to talk, interact, and discuss problems with each other and the therapist. Intensive group therapy provides to the individuals a safe and comfortable place where they can work out problems and emotions^6^. The question of whether the success of the individual intervention could be replicated in a group situation without a reduction in individual effectiveness has been the focus of much research. In intensive group therapy, smoking cessation clinics can provide regular group treatments, and healthcare personnel who are conducting smoking cessation services at the clinics can use their time more efficiently. Intensive group therapy can provide additional help to maintain abstinence in the first month. After the quit attempt, during which most relapse occurs, it can encourage the use of positive smoking cessation strategies. Personnel who conduct the smoking cessation service at the government clinic can encourage therapeutic group processes by increasing group cohesion and enhancing group pressure to maintain abstinence^8^. At the government health clinic, smoking cessation services which are conducted by medical doctors and pharmacist are called quit smoking clinics. In the quit smoking clinics, patients are provided with counselling and pharmacotherapy for smoking cessation. The standard of care at the quit smoking clinics currently is individual therapy (behavioral intervention) with pharmacotherapy. We aimed to investigate the feasibility and efficacy of the group-based therapy for smoking cessation for smokers who wants to quit smoking.

## METHODS

**Figure 1:**
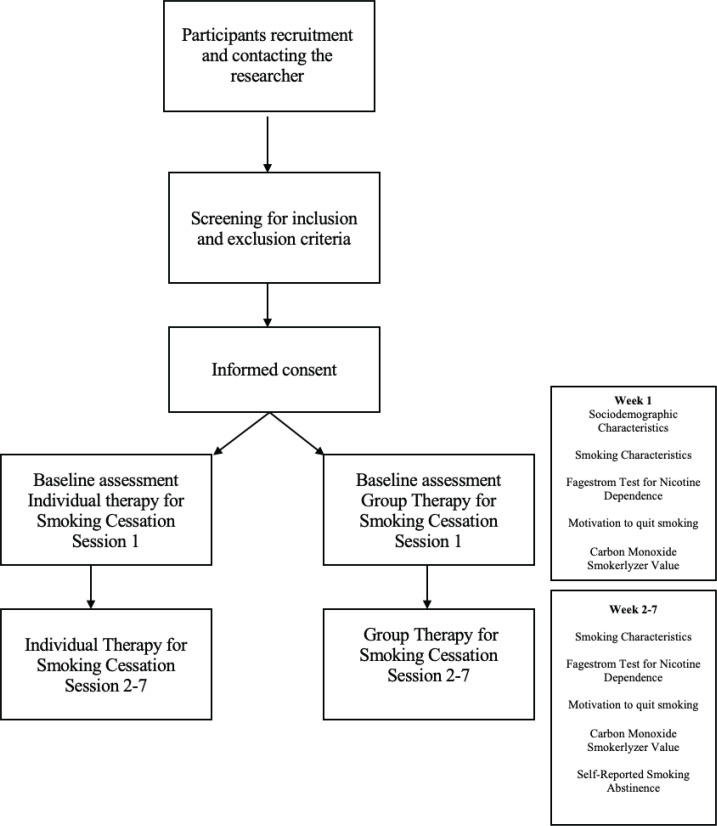
Flow of study

This is a pilot randomized control study conducted among 40 participants (n=20 for individual therapy and n=20 for group therapy) who were active smokers and wanted to quit smoking. Ethics approval was obtained from the Research Ethics Committee, National University of Malaysia Ref No: PPI/111/8/JEP-2020-811 prior to the initiation of the research. Behavioral intervention in the form of group-based therapy and individual smoking cessation was conducted at the Quit Smoking Clinic of the Primary Care Clinic, National University of Malaysia. For feasibility studies, evidence has shown that a sample size calculation may not be appropriate. Active smokers who wanted to quit smoking were invited to join the group therapy for smoking cessation in the general population. Advertisements on social media (such as Facebook, and WhatsApp) were used to recruit the participants to join the intervention. Participants were randomized into individual and group therapy using computer randomization. Participants were eligible to be included in the study if they were active smokers, aged ≥18 years, able to read and understand Bahasa Malaysia or English, able to give consent to participate in the study, and wanted to quit smoking. Participants were excluded if they were aged <18 years, with underlying major mental illness such as schizophrenia and bipolar, were polydrug users, and did not want to participate in the group therapy to quit smoking. The participants were screened for severe mental illness using the Mini-International Neuropsychiatric Interview (M.I.N.I) which is a brief structured diagnostic interview for the major psychiatric disorders used as a screening tool for the 17 most common disorders in mental health. Participants were provided with nicotine replacement therapy (NRT) chewing gum or patch during the intervention. The dosage of the NRT chewing gum was either 2 mg or 4 mg, and 15 mg or 25 mg for the NRT patches, depending on the individual. The flow of the study process is shown in Figure 1.

Due to the nature of the intervention, only single blinding (of participants, not the researcher) was possible. The principal researcher was not blinded to the intervention groups as he was required to know which group each participant has been allocated to, in order to conduct the scheduled follow-up sessions. Group based therapy (GBT) and individual therapy for smoking cessation sessions were conducted by the researcher guided by the smoking cessation module. The module was developed based on the New Zealand smoking cessation module. The module had undergone cultural and linguistic validation for the Malaysian population on each item of the module. The items in the module for week 1 are: introduction to smoking cessation, and information session. The participants who joined the study were introduced to the smoking cessation methods, their motivation to quit smoking was assessed, their level of nicotine dependence evaluated using the Fagerström test for nicotine dependence, and their expired breath carbon monoxide (CO) level measured using a CO Smokerlyzer. On week 2, the participants were prepared for completely abstinence from cigarette smoking and the quit date set. On week 3, the participants were prepared for the quit day by discussing topics related to nicotine withdrawal and how to overcome withdrawal symptoms. From week 4 to week 6 post-quit date sessions, the participants were asked regarding their experiences during the smoking abstinence period and on week 7, participants were given information related to relapse prevention. The items in the module were delivered to 10 participants for the group therapy, and during one-to-one sessions for individual therapy. This provided a direct comparison between individual therapy and group therapy, as the module used for the behavioral intervention has the same content for each week.

During the group-based therapy session, behavioral interventions based on the GBT for smoking cessation module were provided to the participants in 7 weekly sessions and 7 weeks. Due to the COVID-19 pandemic, virtual consultation plus face-to-face consultation were introduced for providing behavioral support to avoid attrition and disruption in the follow-up due to travel restrictions.

During each session, the patients were provided with information, and the discussion for each session based on the suggestion in the module. In the individual group, patients were provided with behavioral support using the standard care practice for individual therapy. Patients in the individual group were followed up weekly for 7 weeks for face-to-face or via virtual consultation. All baseline and follow-up information were assessed during the face-to-face sessions. An outcome assessment was undertaken at the end of the seven sessions. To encourage full follow-up, patients were given an honorarium if they completed all seven sessions.

We aimed to demonstrate the feasibility and efficacy of the group-based therapy for smoking cessation for smokers who wanted to quit smoking by exploring the feasibility and efficacy of the GBT for smoking cessation compared to individual therapy. Outcomes that were evaluated were smoking abstinence at point prevalence (1-week post quit date, week 4; and 1-month post quit date, week 7). In the group-based therapy for smoking cessation module, the quit date was set on week 3. A smoker was counted as a self-reported 4-week abstinent if he/she was assessed face-to-face 4 weeks after the designated quit date and declared that he or she has not smoked even a single puff on a cigarette in the past 30 days. The primary outcome at point prevalence measured was at 4-weeks of smoking abstinence. A smoker was counted as a CO-verified 4-week abstinent if he or she was a self-reported 4-week abstinent (week 7) and his or her expired CO assessed 4 weeks after the designated quit date and found to be <10 ppm (Russell Standard, Clinical). The Russell Standard is a set of criteria widely used to define smoking abstinence. Abstinence is often biochemically verified by measurement of carbon monoxide (CO). The Russell Standard recommends that abstinence should be defined as a self-report of smoking not more than five cigarettes from the start of the abstinence period, supported by a negative biochemical test at the final follow-up^9^. Abstinence from smoking was verified biochemically with a carbon monoxide (CO) concentration value measured by the CO Smokerlyzer. The secondary outcome was 7 days of smoking abstinence after the quit date (week 4). A smoker was counted as a CO-verified 7-day abstinent if he or she was a self-reported 7 days abstinent (week 4) and his or her expired CO was assessed 7 days after the designated quit date and found to be <10 ppm (Russell Standard, Clinical). A similar method was used for individual therapy. Participants who did not attend more than 2 sessions were classified as non-quitters.

Other study data were collected using the questionnaires which were provided to all participants of the group therapy and individual therapy. The data from the questionnaires include sociodemographic characteristics and smoking-related characteristics of the participants, motivation to quit smoking, and level of dependence using the Fagerström test for nicotine dependence (FTND) score. The FTND consists of sets of questionnaires that provide a score of 0–10 with strong test-retest reliability and good validity. High scores indicate intense smokers’ nicotine dependence^9^. Meanwhile, smokers with a higher level of nicotine dependence are less inclined to try to stop and find it more difficult to do so, especially in the early stages^10^. The information was collected from the participants during each weekly session for 7 weeks. The information obtained at week 1 was the baseline and the reported improvement in health status (nicotine dependence, number of cigarettes smoked, CO measurement value) measured at 1 week post quit date (week 4) and 1 month post quit date (week 7) was analyzed. Descriptive and inferential data were analyzed using the SPSS version 27. A p<0.05 for continuous and categorical variables indicates significant statistical differences, and the inferential analysis was 2-tailed.

## RESULTS

Table 1 shows the sociodemographic characteristics of the participants in individual and group therapy using descriptive analysis. The median age of participants was 48 years for individual therapy and 45 years for group therapy, with both groups predominantly of Malay ethnicity. The median monthly income was found to be MYR 1500 (individual therapy) and MYR 2000 (group therapy) (1000 Malaysian Ringgit currently about 210 US$). Participants in both groups smoked about 10 cigarettes per day (median) with the majority of them having at least one attempt to quit smoking. Most of them tried quitting abruptly, and their previous quit attempts lasted at most 12 months for individual therapy groups and 18 months for group therapy groups.

**Table 1:** Sociodemographic characteristics of participants in individual and group therapy in an urban population, Kuala Lumpur, Malaysia (N=40)

	*Individual therapy (N=20)*	*Group therapy (N=20)*
**Age** (years), median	48	45
**Income** (MYR), median	1500	2000
**Race**		
Malay	13	16
Chinese	2	0
Indian	3	0
Other	2	4
**Education level**		
Primary	2	0
Secondary	11	10
Tertiary	7	10
**Number of cigarettes smoked per day** (median)	10	10
**Number of previous quit attempts**		
0	3	3
1	12	12
2	2	4
3	2	0
4	1	1
**The longest duration of the previous quit attempt** (months), median	12	18
**Methods of quitting**		
No quit attempts	3	3
‘Cold turkey’	16	11
Self-help materials	0	1
Individual therapy	1	4
Group therapy	0	1
**Medical illness**		
Diabetes mellitus	4	1
Hypertension	7	4
Cardiovascular disease	1	2

MYR: 1000 Malaysian Ringgit about 210 US$.

### Fagerström test for nicotine dependence score

**Figure 2:**
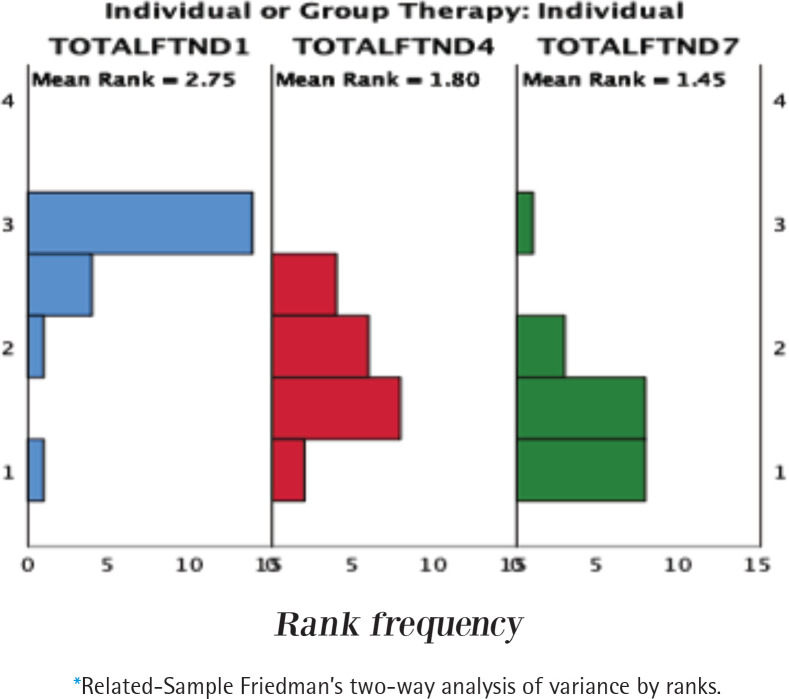
Statistical distribution of data for FTND scores of participants in individual and group therapy following 1, 4, and 7 weeks of treatment

Table 2 shows the median Fagerström test for nicotine dependence (FTND) scores following weeks 1, 4, and 7, in both individual and group therapy. Both groups had improvements in the scores of week one [week 1, median=4 (IQR: 3–6.5)] and one month post quit date [week 7, median=0 (IQR: 0–2.75)] for individual therapy, and week one [week 1, median=5 (IQR: 3.25–5)], and one month post quit date [week 7, median=0 (IQR: 0–2)] for group therapy (p<0.05). However, there was no difference in the scores between weeks 4 and 7 in both groups. On the other hand, the inter-group analysis showed no significant difference in the scores between individual and group therapy groups (p>0.05). Figure 2 shows the statistical distribution of FTND scores for within-group and between-group analyses.

**Table 2:** FTND scores of participants in individual and group therapy following 1, 4, and 7 weeks of treatment, in an urban population, Kuala Lumpur, Malaysia (N=40)

*Therapy*	*Week 1 Median (IQR)*	*Week 4 Median (IQR)*	*Week 7 Median (IQR)*	*Within-group interaction^§^ p*		
				*1 vs 4*	*4 vs 7*	*1 vs 7*
Individual therapy	4 (3–6.5)	1.5 (0–3)	0 (0–2.75)	<0.0001	0.268	0.003
Group therapy	5 (3.25–5)	1.5 (0–3)	0 (0–2)	<0.0001	0.155	0.0003
Between-group interaction* p	0.904	0.841	0.758			

FTND: Fagerström test for nicotine dependence.

* Mann-Whitney U test.

§ Friedman’s test with *post hoc* Bonferroni.

IQR: interquartile range.

### Number of cigarettes smoked per day

**Figure 3:**
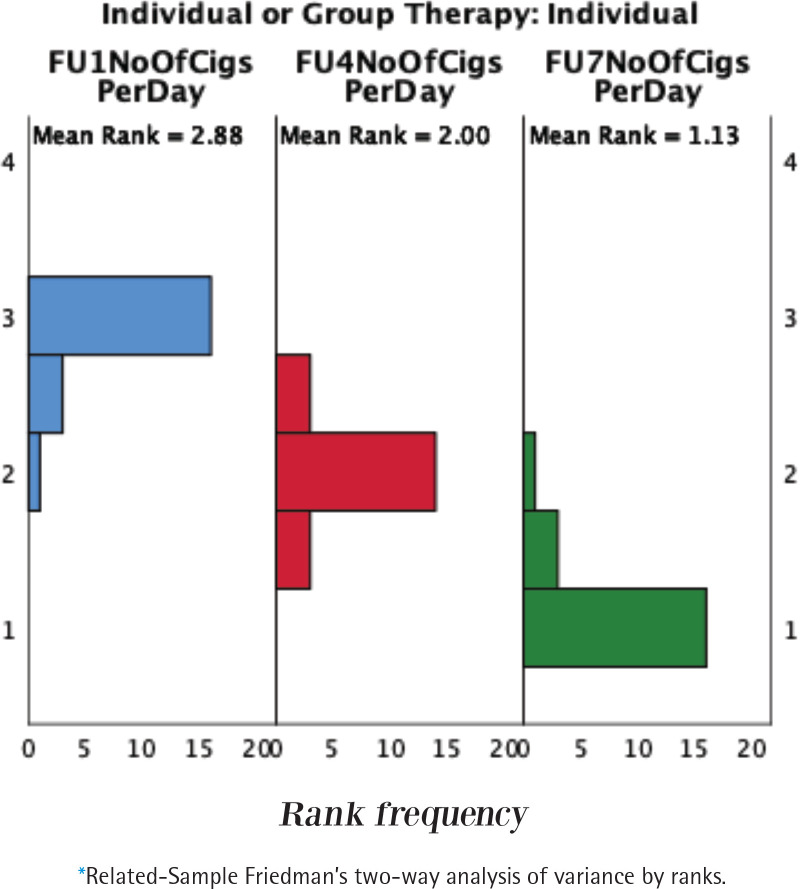
Statistical distribution of data for the number of cigarettes per day of participants in individual and group therapy following 1, 4, and 7 weeks of treatment

Table 3 shows the number of cigarettes smoked per day by participants following weeks 1, 4, and 7, of individual and group therapy. There was a significant difference between weeks 1, and 7 in the median number of cigarettes per day for both individual and group therapy, with a decline in cigarette consumption following the therapies (p<0.05). Both groups had improvements in the scores week one [week 1, median=10 (IQR: 7.75–23.75)] and one month post quit date [week 7, median=0 (IQR: 0–9.5)] for individual therapy, and week one [week 1, median=10 (IQR: 10–15)] and one month post quit date [week 7, median=6 (IQR: 0–9.5)] for group therapy (p<0.05). However, no difference was noted between individual and group therapy (p>0.05). Figure 3 shows the statistical distribution of the number of cigarettes per day for within-group and between-group analyses.

**Table 3:** Number of cigarettes smoked per day by participants in individual and group therapy following 1, 4, and 7 weeks of treatment, in an urban population, Kuala Lumpur, Malaysia (N=40)

*Therapy*	*Week 1 Median (IQR)*	*Week 4 Median (IQR)*	*Week 7 Median (IQR)*	*Within-group interaction^§^ p*		
				*1 vs 4*	*4 vs 7*	*1 vs 7*
Individual therapy	10 (7.75–23.75)	9 (2–10)	4 (0–9.5)	0.006	0.006	<0.0001
Group therapy	10 (10–15)	10 (5–10)	6 (0–9.5)	0.006	0.011	<0.0001
Between-group interaction* p	0.968	0.883	0.799			

* Mann-Whitney U test.

§ Friedman’s test with *post hoc* Bonferroni.

IQR: interquartile range.

### Carbon monoxide level

**Figure 4:**
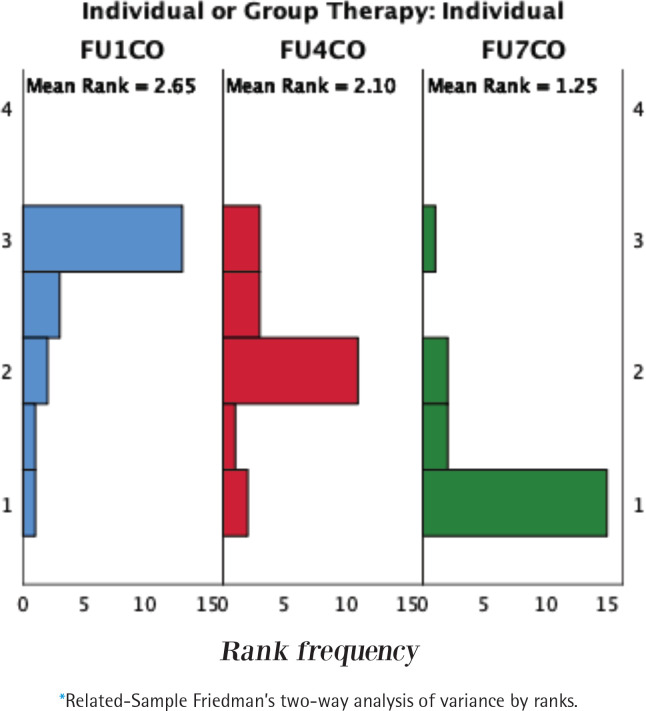
Statistical distribution of data for carbon monoxide level of participants in individual and group therapy following 1, 4, and 7 weeks of treatment

Both groups have shown a decrease in the CO level at weeks 4 and 7 compared to the baseline (p<0.05) (Table 4). This was followed by a significant decline in CO level at week 7 from week 4 in both groups (p<0.05). Both groups have seen improvements in the scores week one [week 1, median=12 (IQR: 6.25–21)] and one month post quit date [week 7, median=3 (IQR: 0.5–10.2.5)] for individual therapy and week one [week 1, median=10 (IQR: 10–15)] and one month post quit date [week 7, median=10.5 (IQR: 8.25–14.75)] for group therapy (p<0.05). However, no significant difference was found in CO level between individual and group therapy at weeks 1, 4, and 7 (p>0.05). Figure 4 shows the statistical distribution of CO levels within-group and between-group analyses.

**Table 4:** Carbon monoxide levels (ppm) of participants in individual and group therapy following 1, 4, and 7 weeks of treatment, in an urban population, Kuala Lumpur, Malaysia (N=40)

*Therapy*	*Week 1 Median (IQR)*	*Week 4 Median (IQR)*	*Week 7 Median (IQR)*	*Within-group interaction^§^ p*		
				*1 vs 4*	*4 vs 7*	*1 vs 7*
Individual therapy	12 (6.25–21)	7.5 (4.25–13.75)	3 (0.5–10.25)	0.082	0.007	<0.0001
Group therapy	10.5 (8.25–14.75)	8 (3.25–12)	5.5 (0–7)	0.006	0.017	<0.0001
Between-group interaction* p	0.547	0.779	0.758			

* Mann-Whitney U test.

§ Friedman’s test with *post hoc* Bonferroni. IQR: interquartile range.

### Current cigarette smoking status

Table 5 shows the percentage of the participants that were actively smoking versus quit smoking in individual and group therapy. There was a steady increase in the number of participants that were not smoking following weeks of treatment. In individual therapy, 5% of participants were not smoking at week 4 and increased to 30% at week 7, while in group therapy, 35% of participants were not smoking at week 7 from 10% at week 4. Between-group analysis showed no significant difference in the number of participants that were not smoking at each respective week between individual and group therapy (p>0.05).

**Table 5:** Current smoking status (% Yes) of participants in individual and group therapy following 1, 4, and 7 weeks of treatment, in an urban population, Kuala Lumpur, Malaysia (N=40)

*Therapy*	*Week 1*	*Week 4*	*Week 7*
Individual therapy	100.0	95.5	70.30
Group therapy	100.0	90.10	65.35
Between-group interaction* p		0.548	0.736

* Chi-squared test.

## DISCUSSION

This study demonstrates the overall effects of smoking cessation through group-based and individual therapy. In general, both group-based and individual therapy have similar efficacy in smoking cessation. The majority of the participants were males of Malay ethnicity, with a median age of 48 years for individual therapy and 45 years for GBT. A Malay male dominance among the participants was found, similar to the race and gender distribution of smokers in a previous study in which Malay males (55.9%) accounted for 59.3 % of the smokers^11^. On the other hand, it is noteworthy to point out that the participants recruited in this study were all males. As expected, as a previous report indicated, of 1989 Malaysians 35.5% of males and only 1.8 % of females were smokers^12^. The monthly median income for participants was in the bottom 40% of the Malaysian income, which agrees with the association of smoking with lower income^13^.

Individual therapy was established as one of the behavioral interventions for smoking cessation with strong evidence reporting its efficacy in numerous randomized controlled trials^3^. Our findings have indicated that GBT, particularly among the Malay ethnicity in the Malaysian population, can increase smoking abstinence without increasing the risk of harm to the patients. To support it, the present findings illustrate several important findings that show the effectiveness of both individual therapy and GBT. It is reflected by the significant findings of the decline, from the assessed parameters from the baseline. Therefore, our findings are in agreement with previous reports in which GBT is equally efficacious as individual therapy.

The current study showed that the FTND score decreases significantly from week one to week four, and then drops to zero on week seven, in both individual and GBT groups. However, the between-group analysis showed no significant findings. Albeit no significant changes were found between the FTND score of individuals and the GBT group, the findings suggest that both types of therapy help reduce nicotine addiction. In both settings of therapy, the smoking abstinence among the participants was reflected by the decline in CO levels in which 30% of participants in individual therapy and 35% in group therapy recorded themselves as not smoking. The present study has several concerns that need to be addressed. The findings demonstrated that there was no significant superiority of group therapy over individual therapy for smoking cessation, although within-group displayed positive results. A real-time systematic review was conducted by Stead et al.^3^ who showed that, over the last two decades, there was a lack of evidence to support the effectiveness of group therapy over individual therapy as a behavioral intervention for smoking cessation. Of sixty-six trials meta-analyzed, six trials compared the efficacy of group therapy versus individual therapy and found no superiority of one therapy over the other (n=980; RR=0.99; 95% CI: 0.76–1.28, I^2^=9%)^3^.

It is worth mentioning that the primary purpose of this study is to elucidate the efficacy and feasibility of group therapy that was adopted from the New Zealand group-based therapy (NZGBT) for smoking cessation protocol in the local settings, instead of demonstrating its superiority over any form of therapy. Given the findings, group therapy (within-group analysis) could provide insight for its future incorporation into the standard of care as an alternative, rather than as a replacement. It is particularly important that patients can choose the behavioral therapy, either to be conducted in a group or individual, whichever best suits them.

Facilitating behavioral change by providing behavioral intervention is given the utmost importance. Smokers were informed that with proper counselling and treatment, their chances of quitting were higher compared to minimal or no intervention. Clinicians, in particular, play an important role in the success of quitting cigarette smoking. Evidence has shown repeatedly that both minimal (<20 minutes in a single visit) and intensive (≥20 minutes for one or more follow-up visits) delivered by clinicians were effective in smoking cessation treatment for at least 6 months^14,15^. Although health facilities provide behavioral support and pharmacotherapy, unfortunately, undertreatment is commonly seen in smoking cessation with relapse occurring up to 50%. Clinicians who were treating patients did not offer counselling or discussion on smoking cessation because they lacked training or they felt they lacked the requisite knowledge to do so effectively^16-18^. As a result, this would further encourage smokers to attempt to quit smoking ‘cold turkey’ by trying to quit smoking unassisted^19^. Interventions which are more widely available and easily accessible, such as minimal intervention for smoking cessation, would be more appealing compared to intensive intervention. Fortunately, this study has shown that group therapy for smoking cessation is an alluring option to assist smokers to quit smoking because of its structured behavioral support provided by trained smoking cessation practitioners. Evidence has shown that a robust intervention provided by a smoking cessation practitioner with more sessions attended by the participants would provide greater success in quitting cigarette smoking. Our study found that it is possible that group therapy for smoking cessation in the Malaysian population further promotes adherence to treatment by addressing withdrawal symptoms, acknowledging mood changes, improving self-efficacy, and learning new skills from others. We have found that this is similar to other studies as reported by Fiore et al.^15^.

The counselling sessions provided the mechanism required for the behavioral change transition from an active smoker to smoking abstinence. The pharmacotherapy, provided for smoking cessation, helps with the withdrawal symptoms when quitting and helps facilitate behavioral change when quitting cigarette smoking. Our study found that it is possible that group-based therapy for smoking cessation in the Malaysian population further promotes adherence to treatment by addressing withdrawal symptoms, acknowledging mood changes, improving self-efficacy, and learning new skills from others. We have found that this is similar to other studies as reported by Fiore et al.^15^.

### Limitations

This study has its constraints, encompassing a restricted sample size and its single-blinded nature. Given that the structure of group therapy can exhibit slight variations across different trials, establishing an unequivocal assessment of group therapy’s superiority over individual therapy proves challenging. The absence of standardized group therapy formats for comparative analysis underscores the novelty of this study, as it aims to evaluate the effectiveness of group therapy for smoking cessation within an urban Malaysian population.

## CONCLUSIONS

This study explored the feasibility of the group-based therapy for smoking cessation that will be useful to reflect the realities in the community and practical setting. The present study identified that group therapy is as effective as individual therapy for smoking cessation. However, further trials investigating the role of group therapy for smoking cessation for the Malaysian population and other Asian countries are warranted.

Group therapy should be easily available and accessible to the general public and can be proposed to be included in the Malaysian clinical practice guidelines for tobacco addiction. Group therapy opens up the possibility for other healthcare practitioners, not only clinicians, to participate in delivering the therapy, as it does not involve an element of pharmacotherapy. Therefore, it can reach a more diverse population as people from non-clinical backgrounds can execute it.

## Data Availability

The data supporting this research are available from the authors on reasonable request.
